# Video-observed treatment for TB patients during COVID-19 in Senegal: efficacy, feasibility, and acceptability

**DOI:** 10.5588/ijtldopen.26.0158

**Published:** 2026-11-13

**Authors:** N.O. Toure, M. Kane, M.F. Cissé, M. Ndiaye, O. Diagne, T.A. Ba, N. Fall, V. Veronese, C.S. Merle, Y.M. Diop, F. Danfakha, B. Gning

**Affiliations:** 1 Centre Hospitalier National Université de Fann, Dakar, Senegal;; 2 Le Programme National de Lutte contre la Tuberculose (PNT) au Dakar Sénégal;; 3 The Special Program for Research and Training in Tropical Diseases (TDR), World Health Organization, Geneva, Switzerland.

**Keywords:** tuberculosis, digital health, digital adherence technology

## Abstract

**BACKGROUND:**

The COVID-19 pandemic caused disruption to TB services and negatively affected key TB control indicators. To support continuity of care and strengthen treatment adherence during the pandemic, a video-observed therapy (VOT) intervention using WhatsApp was introduced and evaluated among TB patients in Dakar, Senegal.

**METHODS:**

A mixed-methods study conducted between January and March 2021 compared adherence, clinical progress, and treatment outcomes among patients receiving daily VOT and those receiving standard, facility-based directly observed therapy (DOT). Feasibility and acceptability were also explored through interviews with patients and TB clinic staff.

**RESULTS:**

Ninety-seven patients received VOT across five clinics, and 146 patients received DOT and formed the comparison group. Participants were predominantly male with a mean age of 32.5 years. At the end of the intensive phase, adherence was slightly higher in the VOT group compared with DOT (78% vs. 73%), and acid-fast bacillus positivity was marginally lower (14% vs. 17%). By treatment completion, cure rates were high and similar in both groups. Qualitative findings indicated that VOT was acceptable and feasible for patients and providers, though challenges included unreliable internet connectivity and concerns about privacy.

**CONCLUSION:**

Overall, VOT appears to be a practical alternative to standard DOT, particularly during public health emergencies that limit access to in-person care.

TB remains a major public health problem in Senegal. In 2020, there were an estimated 20,000 cases of TB; however, approximately 12,000 TB cases were notified nationally during the same period, suggesting that as many as a third of all TB cases are unreported and/or undetected.^1^ Treatment coverage and success rate among new and relapse cases in 2020 were estimated at 65% and 91%, respectively.^1^ Despite the efforts made by the National TB Programme (NTP), national TB elimination targets have not yet been met. In 2020, a new national plan of action was launched to accelerate the fight against TB.^2^ However, the implementation of the new action plan against TB was disrupted by the arrival of COVID-19. The COVID-19 pandemic led to wide-scale disruption of health services worldwide, both in high- and low-resource settings, with TB screening and detection services being among the most disrupted.^3^ The COVID-19 pandemic had a negative impact on TB control indicators, and various measures were implemented by the Senegalese government under a state of emergency decree to limit the spread of the virus. This included restrictions on personal movement and public transport, which also likely impacted treatment-seeking behaviour of individuals with TB symptoms, as has been noted elsewhere in the region.^4–7^

Both generally and in the context of the COVID-19 pandemic, digital tools have shown promise in helping to overcome some of the noted barriers to TB screening and treatment.^8–11^ Digital tools have been deployed as a way to simplify and support the workload of health care workers during COVID-19, by circumventing challenges to patient access to TB services, for example, or as a way to facilitate efforts to boost TB service provision and performance in the subsequent, ‘living with COVID-19’ phase.^8,12^ In particular, there have been specific calls for the adoption of digital tools for TB adherence in the African context, which to date have been under-utilised.^11^

To maintain the continuity of TB treatment and care in the context of the COVID-19 pandemic, a novel intervention to provide virtual directly observed therapy, short-course (VOT) using an online, video-enabled social media platform (WhatsApp) was implemented and evaluated among TB patients in the Dakar region of Senegal.

## METHODS

A mixed-methods study was undertaken to evaluate a VOT intervention implemented in five TB diagnostic and treatment centres (DTCs) across the Dakar region comprising a quantitative, quasi-experimental cohort study with a comparison group and qualitative, key informant interviews with TB patients and DTC staff.

Daily video calls using the WhatsApp platform were made between eligible drug-susceptible TB patients and DTC managers to observe daily TB treatment during the intensive phase of treatment (months 1–2) for patients receiving a standard 6-month regimen. Eligible patients were provided with an advance allocation of TB treatment: weekly allocations were provided during the first month which increased to monthly allocations from month 2 to month 6 (treatment end) for patients demonstrating good adherence (defined as >80% participation in daily video calls). Both TB patients and DTC managers were provided with monthly credit for internet and phone connection during the intervention. VOT patients were also required to present to their managing DTC at months 2, 5, and 6 for clinical monitoring and assessment.

### Outcomes of interest

The study outcomes of interest were effectiveness, feasibility, and acceptability of the digital intervention among TB patients and DTC managers. Effectiveness was collectively defined as: the number of patients attending at least 80% of video calls per month of treatment during the intensive phase; proportion of patients with negative treatment outcomes (those whose treatment outcome was categorised either as death, treatment interruption, or loss to follow-up); and proportion of TB patients cured at the end of treatment (defined as the proportion of patients with bacteriologically confirmed TB at the beginning of treatment who were smear- or culture-negative in the last month of treatment and on at least one previous occasion). Feasibility and acceptability were assessed through quantitative key informant interviews with TB patients and DTC staff to assess attitudes and perceptions of VOT. Feasibility was also assessed via patient adherence – defined as participation in at least 80% of VOT/DOT sessions.

### Participant eligibility and recruitment

For the quantitative study, individuals with bacteriologically confirmed pulmonary TB who were commencing treatment for drug-sensitive TB at the time of the study in any one of the five selected DTCs in the Dakar region, and gave their consent to participate, were considered eligible for the study. Ineligible participants were defined as those with bacteriologically confirmed pulmonary TB patients whose clinical condition required hospitalisation, those with drug-resistant TB, and/or those who refused to participate in the study. Eligible patients were recruited between January and March 2021 using convenience sampling at each of the five DTCs. Study sites were randomly selected from a stratified list of urban, peri-urban, and rural facilitates, respectively. Among the five DTCs selected, two represented urban facilities, two represented peri-urban facilities, and one represented rural facilities. Recruitment continued until a sample of 20 bacteriologically confirmed pulmonary TB patients from each site (total of 100 patients) were enrolled into the intervention group. This sample size was pragmatically selected based on the typical number of eligible patients presenting at the five DTCs during the study duration. All other bacteriologically confirmed pulmonary TB patients who were initiated and followed up by the five DTCs during the study period served as the control group and received the regular standard of care DOT: daily visits for treatment supervision during the intensive phase (months 1 and 2) and the standard of care follow-up by health workers during months 3–6.

For the qualitative component, eligible TB patients were selected from those participating in the quantitative study, with one male and one female patient in the intervention group randomly selected at each site by the study team and invited to participate. CDT managers from the study sites were also included.

### Data collection and analysis

A quantitative survey was developed and administered to TB patients by a member of the research team at CDT sites. Additional data was extracted from patient files related to the management of TB: age, sex, time of VOT, acid-fast bacillus test results at months 2, 5, and 6, and treatment outcome. Patient results were aggregated by intervention and control groups to allow comparison between the two groups.

Qualitative interviews were conducted on the advantages and disadvantages of VOT and recommendations for improving the intervention. A structured interview guide was used by the study team to conduct qualitative interviews by phone with participants. Notes were taken by the interviewer during each call which were aggregated at the end of the study and analysed using a deductive approach to determine the overall level of feasibility and acceptability of VOT among TB patients and CDT managers.

All primary and secondary data collection was done by a member of the research team who was not involved in providing TB care in order to reduce any potential sources of bias.

Descriptive statistics were used to summarise patient characteristics, and differences between the two groups were assessed for significance (using either the Mann–Whitney test, Pearson’s χ², or Fischer’s test as appropriate). The significance level used was α = 0.05.

### Ethical statement

This study received ethical approval from the national ethics committee of Senegal (protocol SEN20/84) in December 2020. Informed consent was sought from all patients who were enrolled in the intervention group to receive VOT. For the other patients who were commenced on treatment during the study period, as only routinely collected and anonymised patient data were extracted from patient files and recorded anonymously, an informed consent waiver was approved.

## RESULTS

### Quantitative findings

In total, 97 patients were enrolled in the intervention group across the five DTC study sites (three patients were excluded after enrolment because they were later identified as having extra-pulmonary TB). An additional 146 other bacteriologically positive TB patients were also enrolled for treatment in the five centres during the study period and acted as the control group, receiving the standard of care DOT. Overall, participants were mostly male with an average age of 32.5 years. Participants in the control group were older on average than those in the intervention group and had a higher male-to-female ratio (Table 1).

**Table 1.** Socio-demographic characteristics of participants (n = 243).

		Total (n = 243)	VOT group (n = 97)	DOTS group (n = 146)	*P* value
Ratio of men to women		2.11	2.03	2.17	0.72**^A^**
Age in years (median age)		32.5 years	30.8 years	33.9 years	0.09**^B^**
<14 years	8 (3.3%)		4 (4.1%)	4 (2.7%)	
15–50 years	210 (86.4%)		86 (88.7%)	124 (85%)	–
>51 years	25 (10.3%)		7 (7.2%)	18 (12.3%)	–

*P* values < 0.05 considered statistically significant.

VOT = video-observed therapy; DOTS = directly observed therapy, short-course.

A Fisher’s exact test (sex ratio).

B Mann–Whitney *U* test (median age).

Among patients receiving VOT, 78.3% (n = 76) met the criteria for adherence (defined as attendance of at least 80% of daily VOT calls) during the first 2 months, or at least 48 of the 60 days. Among patients in the control group, adherence during daily DOT in the first 2 months was 73.3% (Table 2). Adherence in the VOT group declined between months 3 and 5, with 68.0% of patients (n = 66) meeting the 80% attendance requirement (equal to at least 72 of 90 days), with a minimum duration noted of 23 days. Five of the patients who did not reach the minimum number of VOT sessions during months 2–5 reported internet connection problems. Adherence remained stable at month 6, with 67% (n = 65) of patients meeting the 80% required attendance (24 of 30 days) and a minimum noted duration of 12 days (Table 2). The average duration of a VOT session was 2 minutes.

**Table 2.** Proportion of adherence among patients in intervention and control groups.

Period of assessment	Proportion of adherent**^A^** patients – VOT (n)	Proportion of adherent patients – standard DOTS (n)
Months 1–2	78.3% (76)	73.3% (107)**^B^**
Months 3–5	68.0% (66)	Not applicable
Month 6	67.0% (65)	Not applicable

VOT = video-observed therapy; DOTS = directly observed therapy, short-course.

A Adherence defined as participation in at least 80% of daily calls (intervention group) or visits (control group).

B Patients in control group were only required to attend daily, in person DOT visits during the first 2 months of treatment.

All patients underwent clinical monitoring at the end of months 2, 5, and 6. At the end of month 2, 16% of all patients were AFB+, with a slightly greater proportion of AFB+ patients in the control group (17.1% vs. 14.4% in the intervention group). At month 5, only three patients were AFB+ (two in the control group), and by the end of month 6, only one patient in the control group was AFB+. Loss to follow-up was slightly higher in the control group compared to the intervention group (4.8% vs. 3.1% respectively), and one patient in the control group died.

By the end of 6 months, 93.8% of all patients were classified as cured, with a similar proportion of cured patients in both groups (95.9% in the intervention and 92.4% in the control group; Table 3).

**Table 3.** Outcomes of patient monitoring and follow-up during treatment course.

		Total (n = 243)	Virtual DOT (n = 97)	DOT (n = 146)
Month 2	AFB+	39 (16.04%)	14 (14.4%)	25 (17.1%)
	Transferred	1 (0.4%)	1 (1.0%)	0
	Treatment interruption	0	0	0
	Lost to follow-up	0	0	0
	Died	1 (0.4%)	0	1 (0.7%)
Month 5	AFB+	3 (1.2%)	1 (1.0%)	2 (1.4%)
	Transferred	2 (0.8%)	1 (1.0%)	1 (0.7%)
	Treatment interruption	1 (0.4%)	0	1 (0.7%)
	Lost to follow-up	10 (4.1%)	3 (3.1%)	7 (4.8%)
	Died	1 (0.4%)	0	1 (0.7%)
Month 6	AFB+	1 (0.4%)	0	1 (0.7%)
	Transferred	2 (0.8%)	1 (1.0%)	1 (0.7%)
	Treatment interruption	1 (0.4%)	0	1 (0.7%)
	Lost to follow-up	7 (4.1%)	3 (3.1%)	7 (4.8%)
	Died	1 (0.4%)	0	1 (0.7%)
	Cured (*P* = 0.8)	228 (93.8%)	93 (95.9%)	135 (92.4%)

AFB+ = acid-fast bacillus positive; DOT = directly observed therapy.

### Qualitative findings

Overall, 10 patients (two from each centre; 50% male) and 10 CDT managers participated in the qualitative interviews.

### Feasibility

CDT managers felt that the scheduling of daily VOT calls, which took place between 6:30 and 9:00 am each day, was convenient and allowed for a good organisation of workload regarding the management of patients. Managers described some challenges in scheduling VOT calls during the Ramadan period among patients who observed fasting; to address these challenges, CDT managers recommended that patients could record a video of themselves taking treatment before the start of the fasting period (normally around 5 am) which could be shared later that morning with CDT staff for verification. In addition, some technological challenges were reported which compromised the overall feasibility of the intervention, such as smartphones that weren’t working correctly or problems establishing or maintaining an internet connection.

### Acceptability

Qualitative findings pointed to a good level of acceptability of VOT among patients, who expressed their appreciation for the practical nature of the intervention. Some patients however expressed concerns for privacy which appeared to lower the overall level of acceptability of VOT. Some patients – both male and female – noted that they needed to explain the study and its intent to their spouses to minimise any suspicion around the frequent and early morning calls. Additionally, CDT managers noted that two of their patients were university students whose adherence to VOT calls dropped when they returned to school and were required to share a room with other students, suggesting that a fear of disclosure of TB status and its associated stigma and discrimination were barriers to participating in the VOT intervention.

## DISCUSSION

The African region has 16 of the 30 highest TB-burden countries, where TB remains a leading cause of death and the rise of multidrug-resistant TB poses a serious health risk.^13^ Sub-optimal TB treatment adherence contributes to poor treatment outcomes, ongoing transmission, and drug resistance.^13,14^ This study demonstrated that VOT is a feasible, acceptable, and effective tool for supporting TB treatment adherence during the COVID-19 pandemic in Senegal. Our findings suggest that VOT is a useful tool for bolstering treatment adherence in Senegal, particularly during periods where in-person attendance is not feasible as was observed during the COVID-19 pandemic.

The WHO recommends digital technologies – including SMS reminders, electronic monitoring, and VOT – as innovative tools to support treatment for drug-susceptible TB^15^; however, these tools remain underused in Africa despite calls to expand them during the COVID-19 pandemic.^11^ A key advantage of VOT is that it enables remote management of TB treatment and reduces facility visits, saving patients both time and money.^16–20^ Nonetheless, VOT depends on adequate device capacity, connectivity, and user skills.^21^ Although mobile and internet penetration in Senegal is high, our study highlighted that connectivity challenges limited the overall feasibility of VOT, consistent with findings from Uganda showing that poor connectivity and internet access also diminished the overall acceptability of VOT.^18,22^ Despite this, such barriers are likely to decrease as telecommunications infrastructure and investment in Africa improves.^23^

This study found that a lower proportion of VOT patients were AFB+ at follow-up compared to DOT patients, which also points to the role of VOT in supporting treatment adherence. Although between-group differences in AFB positivity did not reach statistical significance (*P* ≥ 0.50), this finding suggests that VOT may help promote adherence and treatment effectiveness and aligns with meta-analysis findings showing higher adherence and slightly higher bacteriologic resolution among VOT users,^21^ as well as other studies that associated VOT with improved treatment completion.^20,24,25^ In addition, both patients and CDT staff in our study appreciated the convenience and time savings of VOT, which has been found to significantly reduce travel time for patients and health care workers compared to traditional DOT.^18–20^ Although cost-effectiveness data from low- and middle-income countries (LMICs) remain limited,^26^ studies in high-income settings have found substantial per-patient cost savings for VOT, largely driven by reduced human-resource needs,^27^ underscoring the potential economic value of VOT and the need for cost-effectiveness studies in LMICs. To our knowledge, this is the first study to assess digital adherence technologies for TB treatment in Senegal, finding that VOT was feasible, acceptable, and effective to TB patients and providers.

Our study was conducted as an implementation research project in the context of the COVID-19 pandemic and has the following limitations: first, given the pragmatic nature of our study, we did not employ formal calculations for sample size or power and our study is likely underpowered for formal hypothesis testing. Second, our study population was selected using convenience sampling, and therefore we cannot exclude selection bias, particularly as patients who took part in VOT were required to give informed consent and may have been systematically more motivated than patients from the control group. Baseline differences in clinical severity were also not assessed, and the extent to which this may cause confounding is unknown. Additionally, participants in the VOT group were slightly younger on average than those in the DOT, and while this difference was not significant, younger participants may be more familiar with digital tools and more motivated to engage with technology-based interventions. This finding suggests that there might be certain types of patients for whom this type of intervention is more acceptable/effective, and that VOT should be considered by the NTP as one tool in a broader toolbox, rather than a one-size-fits-all approach, as per a patient-centred care.

## CONCLUSION

Our findings indicate that VOT is a promising alternative to DOT in Senegal and may offer important insights for other similar low-resource settings. Scaling up VOT with a robust evaluation – including cost analysis – would provide critical evidence that could inform future policy, particularly for maintaining continuity of care during pandemics and other public health emergencies. A follow-up study is currently being conducted in Senegal on the basis of these findings to scale up the availability of VOT along with a robust evaluation to further expand the evidence of this approach in LMICs.
